# First report of a *Brucella inopinata*-like atypical *Brucella* strain isolated from a waxy monkey treefrog (*Phyllomedusa bicolor*) in the Republic of Korea: Case Report

**DOI:** 10.3389/fvets.2026.1814429

**Published:** 2026-07-21

**Authors:** Myeongsu Kim, Zun Zun Wut Hmohn, Jae-Ik Han

**Affiliations:** 1 Laboratory of Exotic and Wildlife Medicine, College of Veterinary Medicine, Jeonbuk National University, Iksan, Republic of Korea; 2 Jeonbuk Wildlife Center, Jeonbuk National University, Iksan, Republic of Korea

**Keywords:** atypical *Brucella*, *Brucella inopinata*-like strain, frog, phylogenetic analysis, whole genome sequencing

## Abstract

Atypical *Brucella* spp., which include *Brucella inopinata* and related lineages, have emerged as unconventional pathogens with an expanding host range, particularly among exotic amphibians. A captive waxy monkey treefrog (*Phyllomedusa bicolor*) presented with a focal ulcerative skin lesion above its right eye. Cytology revealed mixed inflammation associated with a bacterial infection, and 16S rRNA sequencing identified the isolate as a member of the *Brucella* genus. Whole-genome sequencing using hybrid PacBio–Illumina platforms generated a high-quality draft genome. Average nucleotide identity (ANI) and digital DNA–DNA hybridization (dDDH) values demonstrated the highest similarity to *B. inopinata* (98.08% ANI; 83.50% dDDH). Core genome phylogenetic analysis further confirmed close clustering with atypical *Brucella* spp., which are clearly distinct from classical zoonotic lineages. This case represents the first detection of an atypical *B. inopinata*-like *Brucella* strain in an amphibian in the Republic of Korea.

## 1 Introduction

*Brucella* spp. are facultative intracellular bacteria that cause brucellosis in a wide range of warm-blooded mammals, including livestock and humans (1). Classical *Brucella* spp. including *Brucella abortus*, *B. melitensis*, *B. suis*, and *B. canis* are well-recognized causative agents of brucellosis. Brucellosis typically manifests with non-specific systemic symptoms such as fever, sweating, and myalgia. This disease is transmitted through direct contact with infected animals or their secretions, consumption of unpasteurized dairy products, and exposure to contaminated water, soil, meat, or other environmental sources. Since *Brucella* spp. can persist within host cells and cause chronic infections, treatment often requires prolonged, multi-drug regimens to achieve complete resolution (2). In the Republic of Korea, outbreaks and clinical cases have been predominantly associated with *B. abortus* and *B. melitensis*, both of which can infect humans (3).

In recent years, a growing number of genetically divergent and ecologically distinct *Brucella* strains, collectively referred to as atypical *Brucella* spp., have been identified. These strains do not belong to the classical mammalian pathogenic species (4, 5). While classical *Brucella* spp. show well-defined host specificity and are primarily associated with warm-blooded mammals, atypical *Brucella* spp. exhibit a much broader host range with diverse environmental niches and cold-blooded hosts, including amphibians, reptiles, fish, and soil (6). Amphibians, particularly frogs, have increasingly been recognized as key reservoirs of atypical *Brucella* spp. (7–11). The isolation of *B. inopinata*, a species capable of infecting humans, from frogs suggests that these amphibians may represent a newly emerging source of potential public health risk (9, 10).

Genetic analysis of *Brucella* spp. is particularly challenging because the genus exhibits remarkable genomic homogeneity (12). This high level of homogeneity has fueled long-standing debates not only about species delineation within *Brucella* spp., but also about its taxonomic distinction from closely related genera such as *Ochrobactrum* spp. Recent genome-based taxonomic revisions have proposed the inclusion of former *Ochrobactrum* spp. within an expanded *Brucella* spp., although this reclassification remains debated (13). Novel *Brucella* spp. continue to be delineated based on their molecular characteristic, host preference, and pathogenicity (14). However, the limited genetic diversity becomes even more problematic when distinguishing atypical *Brucella* spp., making whole-genome approaches such as average nucleotide identity (ANI), DNA–DNA hybridization (DDH), and core genome phylogeny essential for classification (6, 12). ANI provides a quantitative measure of mean nucleotide-level similarity between genomes and is widely used to define species boundaries, with a ≥95%–96% threshold generally indicating the same species (15). DDH, the gold standard for bacterial species delineation, evaluates the extent of hybridization between genomic DNA strands and remains a valuable reference point, with a ≥70% similarity supporting species-level relatedness (16). Core genome phylogeny, which infers evolutionary relationships based on the shared set of conserved genes across strains, has proven to be a robust approach for identifying fine-scale evolutionary differences within the genus (17). These whole-genome approaches provide the resolution needed to distinguish atypical *Brucella* spp. and define their taxonomic position.

There has been increasing research on atypical *Brucella* spp., yet no such pathogens have been detected in the Republic of Korea. This case represents the first report in the Republic of Korea of an atypical *Brucella* spp., specifically a *B. inopinata*-like strain isolated from skin lesions on an exotic frog (*Phyllomedusa bicolor*).

## 2 Case description

**Figure 1:**
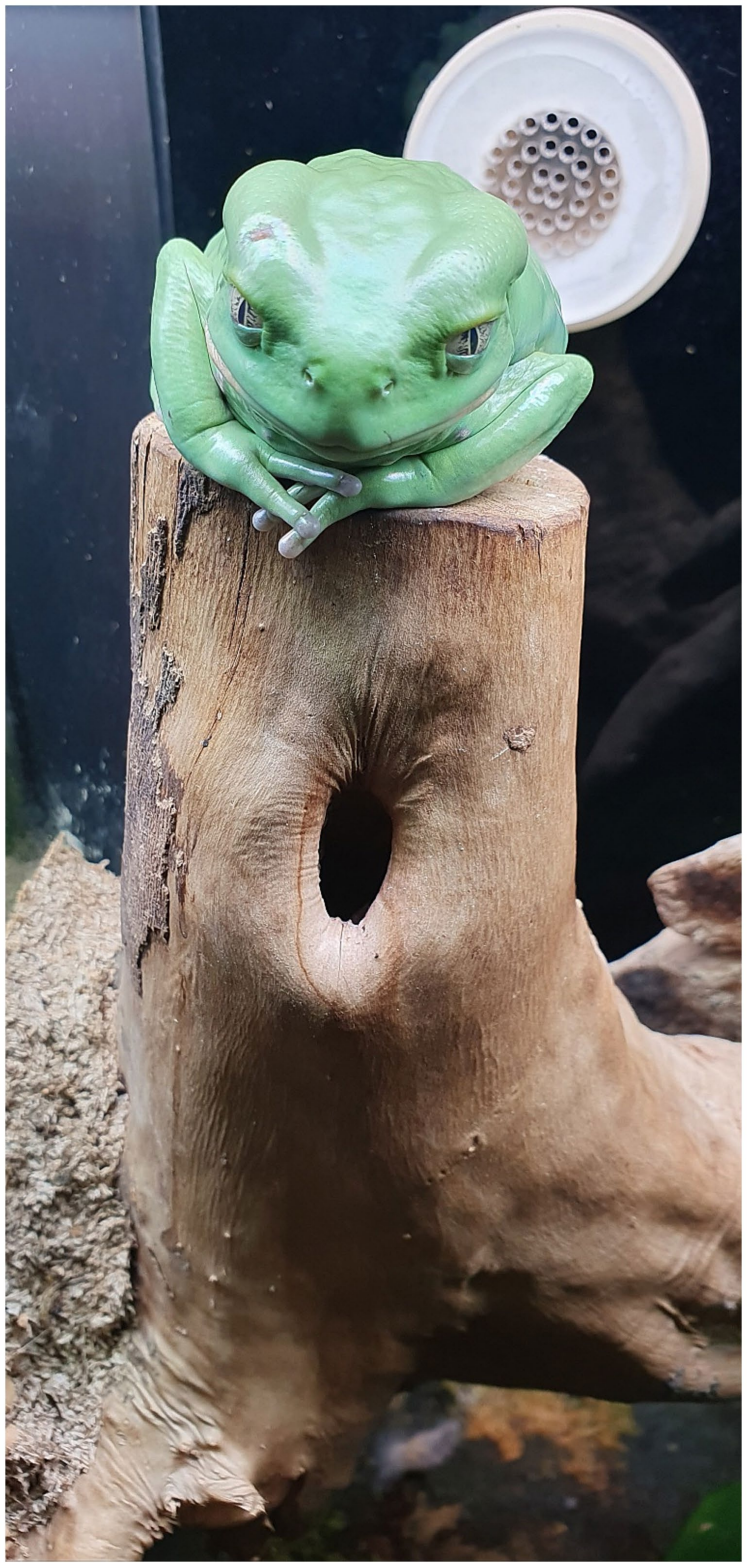
Full body view of the waxy monkey treefrog (*Phyllomedusa bicolor*).

An adult captive waxy monkey treefrog (*P. bicolor*) was referred to the university hospital (Figure 1). Physical examination suggested that a focal ulcerative skin lesion on the dorsal aspect of the right eye had progressed, exposing the underlying muscle. Cytology of the lesion revealed mixed-type inflammation associated with bacterial infection, and bacterial culture and antibiotic susceptibility testing were subsequently conducted. Based on 16S ribosomal RNA gene sequencing results, bacteria isolated from the ulcer were identified as *Brucella* sp. The pathogen was considered susceptible to doxycycline based on large inhibition zones of 32.1 mm, and the animal was treated accordingly. After 1 month, the lesion showed a noticeable reduction in size, and by 3 months, it had diminished to a level consistent with what we expected under spontaneous healing.

**Figure 2:**
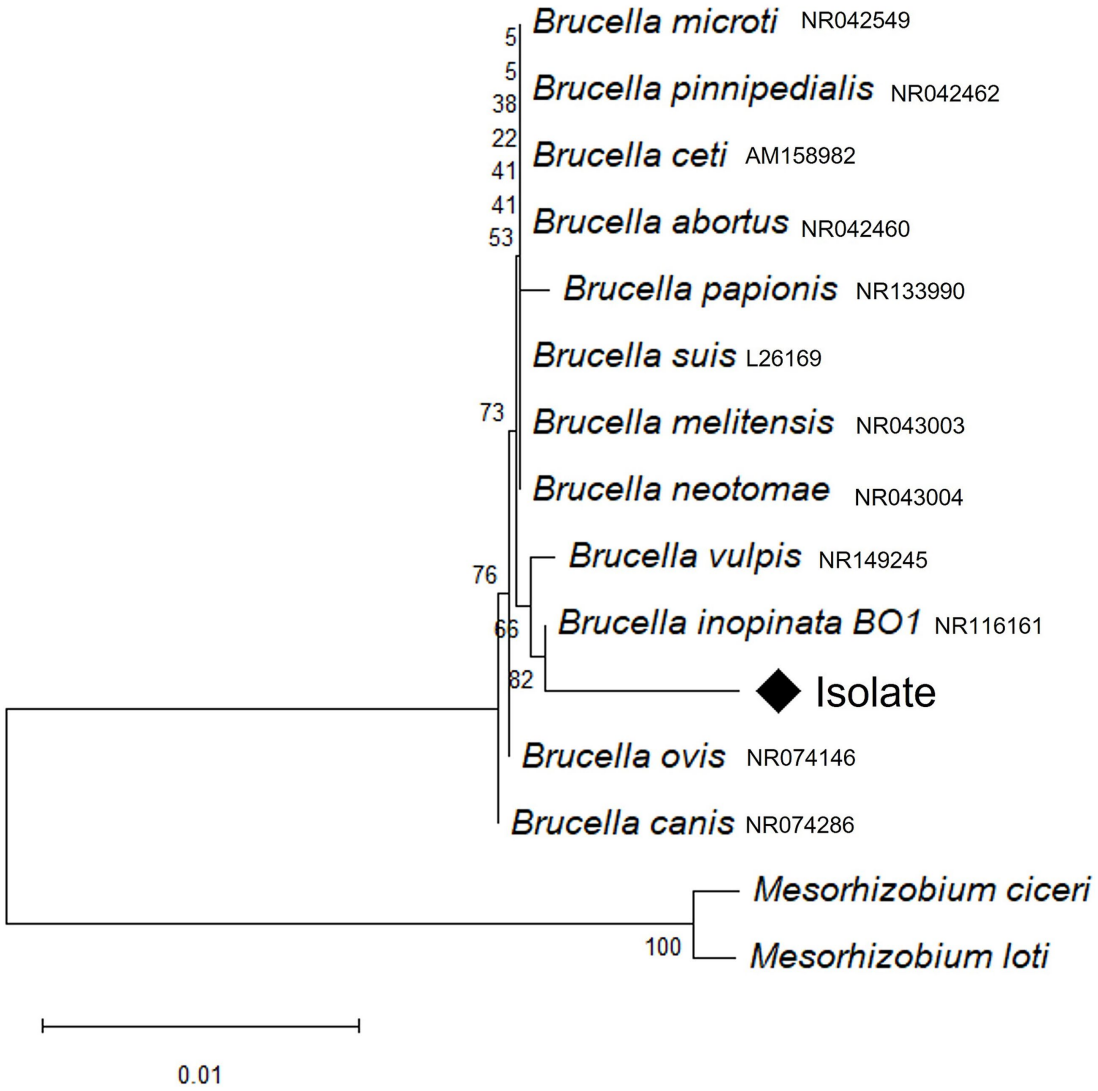
Neighbor-joining phylogenetic analysis based on 16S ribosomal RNA gene sequences in the GenBank database. A black diamond (◆) indicates the *Brucella* sp. identified in the waxy monkey treefrog (*Phyllomedusa bicolor*) in this study. Sequence alignments were performed using ClustalX version 1.8 (34); MEGA-X version10.1 (35) was used for phylogenetic analyses. Bootstrap percentage values are shown on the branches.

To determine the taxonomic position of *Brucella* sp. isolated in this case, phylogenetic analysis was conducted (Figure 2). A neighbor-joining tree based on 16S rRNA gene sequences demonstrated that the isolate clearly belongs to *Brucella* spp. The isolate was clustered most closely with atypical *Brucella* spp., including *B. inopinata*. In contrast, classical zoonotic *Brucella* spp. such as *B. abortus*, *B. melitensis*, and *B. suis* form a separate branch. These findings suggested that the isolate in this case was more closely related to atypical *Brucella* spp. than to classical pathogenic species.

Whole-genome sequencing of the isolate was performed using a commercial hybrid sequencing service provided by Ebiogen Inc. (Seoul, Republic of Korea) using combined PacBio and Illumina platforms. The resulting high-quality reads were assembled and curated to generate a draft genome suitable for genomic analyses. *De novo* assembly and error correction were performed using the Microbial Genome Analysis application implemented in SMRT Link v13.1.0.221970 following the manufacturer’s recommended workflow (18). Hybrid assembly produced two contigs (3.37 Mb; N50 = 2.13 Mb), and quality metrics, including a 99.99% mapping rate and 99.19% BUSCO completeness, confirmed the high accuracy and completeness of the genome, indicating that downstream comparative analysis was appropriate. Assembly validation was performed using Jellyfish, GenomeScope, Inspector, pbmm2, Pilon, BUSCO, and BLAST (19–24). More than 3,000 coding sequences were predicted and functionally annotated using Prokka (25), with functional annotations further refined using EggNOG (26) and InterProScan (27).

Subsequent genomic analyses were conducted to determine the genomic relatedness of the isolate to other *Brucella* spp. ANI was assessed using the OrthoANIu algorithm implemented in EZbioCloud (15). Genome assemblies of representative *Brucella* spp. had ANI values ranging from 97.50 to 98.08%. The highest ANI value was observed in *B.* spp. BO2 (98.10%), *B. inopinata* (98.08%), and *B*. spp. BO3 (97.92%), atypical *Brucella* species, whereas slightly lower ANI values were observed with classical *Brucella* spp. *B. microti* (97.87%) and *B. canis* (97.76%). These results indicated that the isolate in this study was most similar to atypical *Brucella* spp. at the genomic level rather than to classical pathogenic species.

Digital DDH (dDDH) was performed using the Genome-to-Genome Distance Calculator to evaluate genomic relatedness between the isolate and *Brucella* spp. (16). The isolate showed the highest similarity to *B*.spp. BO2 (83.80%), *B. inopinata* (83.50%), *B*. spp. BO3 (83.10%), followed by *B. microti* (81.90%), while classical zoonotic species such as *B. abortus*, *B. melitensis*, *B. suis*, *B. canis*, and *B. ovis* showed slightly lower values (79.30–81.20). These results indicated that the isolate was more closely related to atypical *Brucella* spp. at the genomic level.

**Figure 3:**
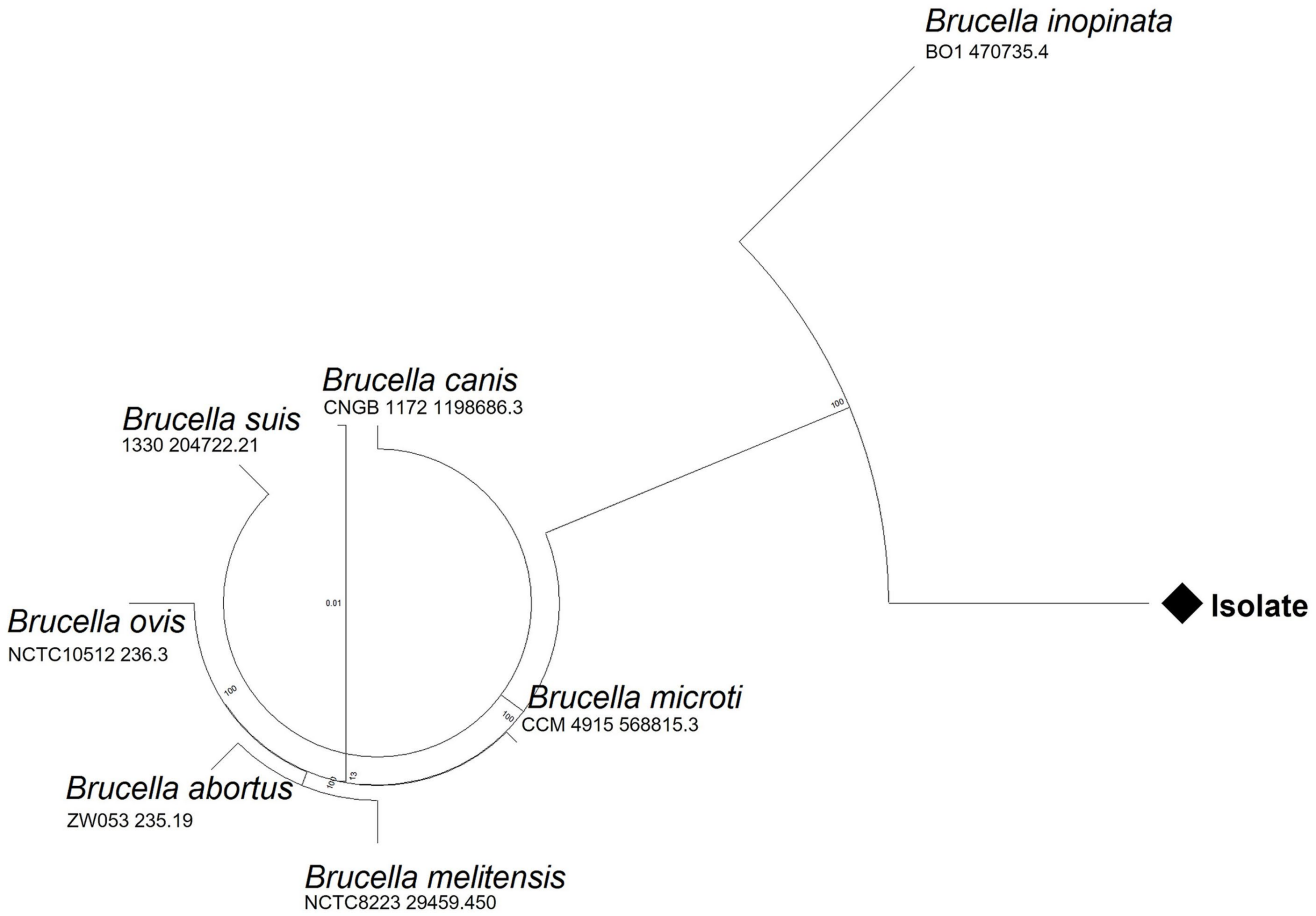
Core genome-based phylogenetic relationships among representative *Brucella* spp. and the isolate (◆) obtained in this study, generated using the Codon Trees pipeline of the Bacterial and Viral Bioinformatics Resource Center. The tree indicated a distinct separation between classical zoonotic *Brucella* spp. (*B. abortus*, *B. melitensis*, *B. suis*, *B. canis*, and *B. ovis*) and atypical *Brucella* lineages, with the isolate clustering closely with *B. inopinata*.

Core genome-based phylogeny was reconstructed using the Codon Trees pipeline at the Bacterial and Viral Bioinformatics Resource Center (28). The resulting phylogeny revealed a distinct separation between classical pathogenic *Brucella* spp. (*B. abortus*, *B. melitensis*, *B. suis*, *B. canis*, and *B. ovis*) and atypical *Brucella* spp. (Figure 3). The isolates in this study clustered strongly with *B. inopinata* and were clearly separated from the classical *Brucella* spp. clade. This core-genome phylogeny corroborated the ANI and dDDH results, indicating that the isolate was most closely related to atypical *Brucella* spp. at the genomic level. Whole-genome sequence-based phylogenetic analysis clearly demonstrated that the isolate was most closely related to *B. inopinata*, supporting its classification within the atypical *Brucella* group.

## 3 Discussion

Newly reported atypical *Brucella* spp. identified across diverse host species have emerged as a potential public health concern (7). Over the past decade, these non-classical *Brucella* spp. have been isolated from mammals (including humans), fish, amphibians and even environmental sources, suggesting that their distribution and host adaptability may be far wider than previously recognized (6, 10). Atypical *Brucella* spp. show high genomic homogeneity, which necessitates whole-genome sequencing for accurate identification; misclassification can lead to clinical issues in both human and veterinary medicine (5, 12).

In this case, whole-genome sequencing-based phylogenetic analysis revealed that the isolate clustered closely with *B. inopinata* and related atypical *Brucella* spp., with the closest relationship observed with BO2, supporting its classification as a *B. inopinata*-like organism. *Brucella inopinata*-like strains have been isolated from humans presenting with brucellosis-like symptoms. Reported cases exhibited clinical signs such as fever, fatigue, and osteoarticular lesions, or, in more severe cases, polyadenopathies accompanied by multiple pulmonary consolidations, and were generally acquired through exposure to infected animals (29, 30). Reported human cases highlight their zoonotic potential, yet atypical *Brucella* spp. often evade classical diagnostic expectations, leading to delayed recognition and prolonged treatment (1, 5, 29). Atypical *Brucella* spp. are particularly concerning because they exhibit marked genomic homogeneity, making them difficult to distinguish from one another and from classical *Brucella* spp. using conventional diagnostic methods (12). It has been suggested that unique traits from atypical *Brucella* spp. could be transferred to classical species through mobile genetic elements, posing a public health concern (1). New genotypes and atypical strains continue to be discovered across diverse hosts and environments, indicating that the diversity of this group remains underrecognized. Such findings heighten the potential zoonotic risk, as genetically cryptic strains may circulate undetected and enter human or animal populations (6, 7, 10).

Although atypical *Brucella* spp. have been detected in a variety of animal hosts, the repeated isolation of *B. inopinata*-like strains from frogs has attracted particular attention (5, 10, 29). In frogs, infections caused by *B. inopinata* and *B. inopinata*-like strains have been associated with clinical signs such as lethargy and skin lesions, and have been isolated from frogs found dead without prior clinical observations (5, 31, 32). Given that *B. inopinata*-like strains have also been recovered from humans with brucellosis-like illnesses, individuals who handle amphibians such as veterinarians, animal keepers, and exotic-pet industry workers may be at increased risk of exposure. The recurrent detection of *B. inopinata*-like strains in anurans suggests that frogs may serve as potential reservoirs or transmission intermediaries for atypical *Brucella* spp. (8, 10, 11).

In this case, a *B. inopinata*-like *Brucella* strain was isolated from a skin lesion of a waxy monkey treefrog (*P. bicolor*). The *B. inopinata*-like strain was rapidly identified from the skin lesion, enabling timely treatment and recognition of the potential risk of transmission to humans or other animals. Antimicrobial susceptibility testing further allowed the initiation of appropriate antimicrobial therapy, and the affected frog ultimately recovered. However, as with classical pathogenic *Brucella* spp. infections, treatment of atypical Brucella spp., including *B. inopinata*-like strains, often require prolonged management (2). In this case, full recovery was achieved only after approximately 3 months. This outcome showed that the possibility of transmission to other animals or humans cannot be fully excluded even when appropriate precautions are followed. The difficulty of determining the timing and route of exposure increases when infected animals are found dead. The fact that *B. inopinata*-like strains can cause disease in humans indicates that individuals working with frogs should exercise caution (29).

In the Republic of Korea, surveillance and research on *Brucella* spp. infections have focused primarily on classical pathogenic species that affect livestock and humans. The potential risks associated with atypical *Brucella* lineages have not been previously recognized (3). This lack of recognition is concerning because atypical *Brucella* spp. display substantial genomic diversity and can infect a broad range of non-traditional hosts, including amphibians. The Republic of Korea has seen a steady increase in the importation of exotic frog species. This increased movement of amphibians across borders poses the risk of introducing novel or previously unrecognized pathogens into the country (33). As the importation of exotic frogs into the Republic of Korea continues to increase, awareness and monitoring of the potential risks associated with atypical *Brucella* spp. are warranted.

In this case, we report the first identification of atypical *Brucella* spp. in an amphibian in the Republic of Korea. This finding highlights the need to broaden national surveillance beyond classical pathogenic *Brucella* spp. and to apply genomic methods that can distinguish atypical strains. The detection of this organism in an exotic amphibian also raises the possibility of spillover to other captive animals, native wildlife, and people who handle amphibians. This case emphasizes the importance of recognizing atypical *Brucella* spp. as emerging pathogens and of implementing careful monitoring to reduce public and animal health risks.

## Data Availability

The 16S rRNA gene sequence generated in this study was deposited in the NCBI GenBank database under accession number PX945274.1. Other datasets used and/or analyzed during the current study are available from the corresponding author upon reasonable request.
